# Anthropometric measurements of non-arthritic knees in an Egyptian population: an MRI-based study

**DOI:** 10.1186/s13018-021-02708-8

**Published:** 2021-09-08

**Authors:** Mohammad Kamal Abdelnasser, Ahmed A. Khalifa, Micheal Bassem, Mohammed Anter Abdelhameed, Mahmoud Faisal Adam, Hatem M. Bakr, Yaser E. Khalifa

**Affiliations:** 1 Orthopaedic and Traumatology Department, Assiut University Hospital, Assiut, Egypt; 2 Orthopaedic Department, Qena Faculty of Medicine and University Hospital, South Valley University, Qena, Egypt

**Keywords:** Anthropometric, Total knee arthroplasty, Arab, North African

## Abstract

**Background:**

Knee anthropometric characteristics were evaluated for different ethnicities; however, data from North African populations are deficient. The primary aim was to investigate the Egyptian knees’ anthropometric characteristics as a representative of North African populations. Secondary aims are as follows: (1) to study the anthropometric gender difference, (2) to compare results with other ethnic groups, and (3) to study the mismatch in comparison to geometric characteristics of modern TKA implant designs.

**Methods:**

Two hundred normal knee MRI scans (100 females and 100 males, aging from 18 to 60) were obtained for analysis. Linear measurements (anteroposterior (AP), mediolateral (ML), and aspect ratio (AR)) of the planned cut surface of the distal femur (f) and the proximal tibia (t) were evaluated.

**Results:**

A significant difference between both sexes was found, males had larger measurements in anteroposterior [fAP: 60.97 ± 3.1 vs 54.78 ± 3.3 (*P* < 0.001), tAP: 46.89 ± 3.0 vs 41.35 ± 2.9 (*P* < 0.001)] and mediolateral [fML: 74.89 ± 3.2 vs 67.29 ± 3.7 (*P* < 0.001), tML: 76.01 ± 3.0 vs 67.26 ± 3.2 (*P* < 0.001)], the mean femoral and tibial AP and ML measurements were different from other ethnic groups. None of the seven studied TKA systems matched the largest ML or the smallest AP dimensions of the distal femur in the current study population.

**Conclusion:**

A significant difference was found between males’ and females’ knee anthropometric characteristics. Some of the commonly used TKA implants in our area could not provide a perfect fit and coverage.

**Trial registration:**

ClinicalTrials.gov identifier: NCT03622034, registered on July 28, 2018.

## Background

Given the fact that total knee arthroplasty (TKA) is a highly successful procedure with documented success to relieve pain and improve motion in patients with end-stage arthritis [1], however, a substantial number of patients are not satisfied after TKA [2], several factors are blamed [3, 4], of these, is the use of an appropriate implant size which maximizes bony coverage and reduces overhang [5].

The different ethnicities’ normal knee anthropometric data helped designing knee implants that provide the perfect size match with a consequent reduction in implant-sized mismatch-related complications [6–10]. Modern TKA prosthesis systems provide incremental femoral and tibial component sizes to offer the best fit for the replaced surface; however, most of these designs are based on measurements driven from white Western males [11–14]. Mahfouz et al. compared the non-arthritic knee anthropometric measurements between three different ethnicities (Asian, Caucasian, and African American) and found a significant difference in the knee dimensions [15]. A similar finding was reported in a systematic review by Kim et al. [16]. Further studies reported differences between various ethnic groups, including Caucasian [17], Indian [18], Thai [19], Korean [20], Chinese [21], Hispanic [22], and African Americans [15]. No similar studies existed for the Middle Eastern or African population [16].

Only one study by Hafez et al. [23] reported the anthropometric details of the Arabian knee; however, their study was based on arthritic knees, which may influence the accuracy of the measurements due to the presence of subchondral sclerosis, osteophytes, and bone attrition which may alter the normal anatomy of the knee, thereby changing the normal knee anthropometry [24].

This study’s primary objective was to define the anthropometric measurements of the non-arthritic Egyptian knee (representing North African populations). The secondary objectives were (1) to study the anthropometric gender difference in our population, (2) to compare our results with other ethnic groups, and (3) to study the mismatch between the current study knee anthropometric values and the geometric characteristics of commonly used modern TKA implants.

## Methods

This is a single-center observational cross-sectional study. Normal magnetic resonance imaging (MRI) scans (as reported by a senior radiologist) performed for patients with suspected knee ligamentous injury were used for analysis after our hospital’s ethical committee’s approval. Adult patients between 18 and 60 years were included. Knees where the MRI shows signs of osteoarthritis, gross bony, or cartilaginous defects were excluded. MRI of 100 males and 100 females was used for this study.

### Imaging technique

A Siemens 1.5 Tesla magnet (Siemens, Erlangen, Germany) and a knee joint surface coil were used for examination. Images were taken at an intervening interval of 0.3 mm and a thickness of 3 mm. To obtain an axial image parallel to knee joints, scans were performed while the patient was lying supine, and the knee was fully extended, keeping the patella towards the ceiling. The obtained images were processed via the local picture archiving and collecting system (PACS) used in our hospital, and the PACS software (Paxera Ultima 360) was used to do the measurements. To ensure the accuracy of the measurements, two of the authors measured each of the endpoints independently, and the average of both measurements was used for final analysis. Measurements were performed as follows:

#### Distal femoral (f) measurement (Fig. 1)

**Fig. 1:**
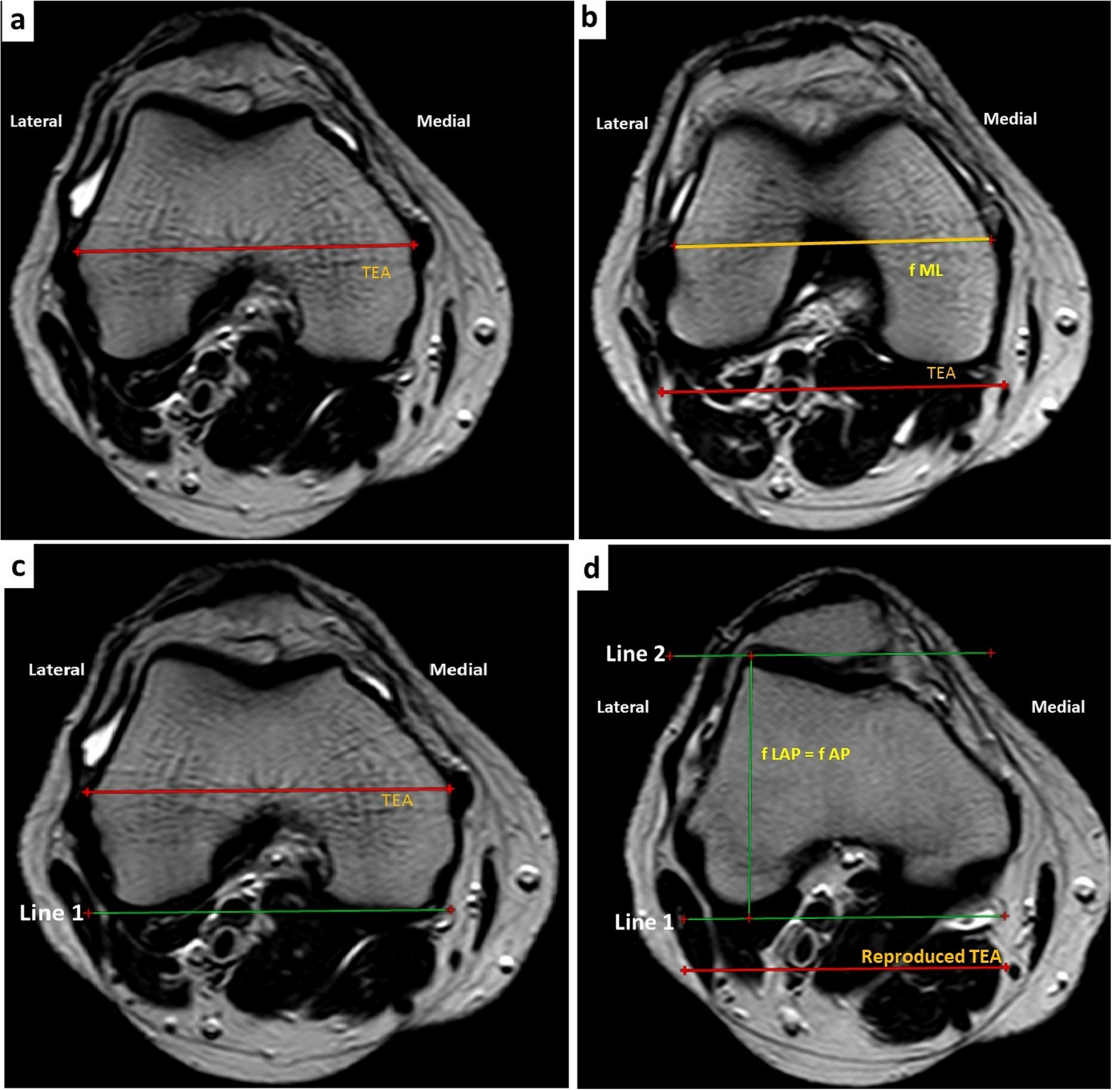
Distal femoral measurements. **a** Identification of the trans-epicondylar axis (TEA). **b** Measuring the femoral mediolateral (fML) length. **c** Line 1 is drawn tangential to the lowest point of the lateral femoral condyle and parallel to the TEA in a corresponding axial cut. **d** Line 2 is drawn tangential to the highest point of the lateral distal femoral condyle (LDFC) and parallel to the TEA, measuring the femoral anteroposterior (fAP) length as the distance between Line 1 and Line 2

The trans-epicondylar axis (TEA) was first drawn as a line connecting the apexes of the medial and lateral femoral epicondyles in the widest axial cut of the distal femur (which contains the epicondyles) (Fig. 1a) [17]. The distal femoral measurements were done in the axial cut 9 mm above the most distal point of the medial femoral condyle to mimic the distal femoral cut in TKA as follows:

1. *The femoral mediolateral (fML) length* was measured as the longest line connecting the medial and the lateral dimensions parallel to the trans-epicondylar axis (Fig. 1b).
2. *The femoral anteroposterior (fAP) length:* according to Kim et al. [16], there is no significant difference between Lateral femoral anteroposterior (fAP) length and the fAP length, so we considered the former as the AP femoral length. As the highest and the lowest points of the lateral distal femoral condyles (LDFC) could not be visualized in the same axial cut, so, we draw a line tangential to the lowest point of the lateral femoral condyle and parallel to the TEA in the corresponding axial cut (Line 1) (Fig. 1c). We reproduced line 1 in the axial cut that contains the highest point of the LDFC, and a second line tangential to the highest point and parallel to the TEA was drawn (Line 2). The vertical distance between Line 1 and Line 2 represents the fAP length (Fig. 1d).
3. *The distal femur’s aspect ratio (AR)* was then calculated as (fML/fAP).

#### Proximal Tibial (t) measurement (Fig. 2)

The femoral TEA was reproduced in the tibial cuts. The proximal tibial measurements were performed in the axial cuts 8–9 mm distal to the joint surface (3 slices from the joint surface) as follows:

1. *The tibial mediolateral (tML) length*: it was measured as the longest mediolateral line (which is parallel to the TEA) in the axial cuts of the proximal tibia [17, 25, 26].
2. *The tibial anteroposterior (tAP) length:* at the same level of the cuts, it was measured as the length of a line drawn perpendicular to the tML through the midpoint of the axial cut (17, 20, 25).
3. *The tibial AR* was calculated as (tML/tAP).

**Fig. 2:**
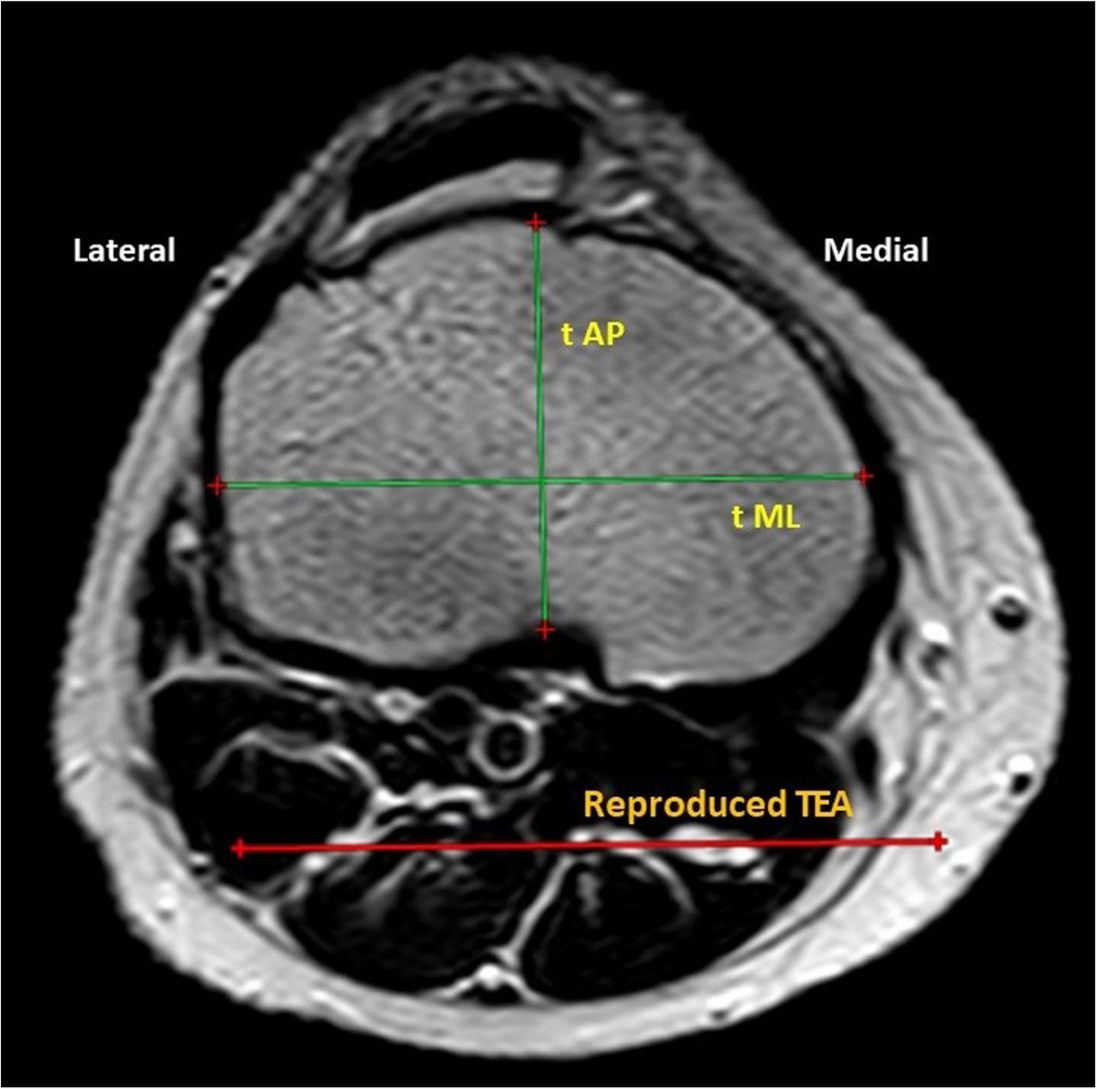
Proximal tibial measurements: The tibial mediolateral (tML) length as the longest mediolateral diameter, the tibial anteroposterior (tAP) as the length of a line drawn perpendicular to the tML through the midpoint of the axial cut. (TEA, trans-epicondylar axis)

Anthropometric measurements of males and females from the current study were compared to detect gender differences in the Egyptian population. These values were then compared with corresponding values for other ethnic groups and with geometric values for modern TKA implants and knee prostheses that are commonly used in Egypt.

Seven implant types were used for comparison. NexGen (Zimmer), PFC-Sigma (DePuy, J & J), Scorpio PS (Stryker), Genesis 2, Journey 2, and Anthem (Smith & Nephew), and Freedom (Maxx Health). The first three implants are widely used in our country. Scatter graphs were plotted with the patient size and the best possible implant size for all the implants.

### Statistical analysis

Data were verified, coded by the researchers, and analyzed using SPSS (IBM_SPSS. Statistical Package for Social Science. Ver.21. Standard version. Copyright© SPSS Inc., 2011–2012. NY, USA. 2012.). Descriptive statistics: Means, standard deviations, and ranges were calculated. Intraclass Correlation Coefficient (ICC) was used to test the inter-rater reliability (ICC > 0.5 is considered acceptable, > 0.6 moderate, > 0.7 good, > 0.8 high, and > 0.9 excellent). Test of significances: for continuous variables, a one-sample *t* test was used to compare the mean of the study parameters against each of the other studies’ single mean. Independent *t* test analysis was carried out to compare the means of normally distributed data, while the Mann-Whitney *U* test was calculated to test the median differences of the data that do not follow a normal distribution. A *p* value **≤** 0.05 was considered significant.

## Results

The inter-rater agreement (ICC) for all measurements was > 0.8. The study population's mean age was 34.16 ± 11.24; there was no difference between males and females (32.12 ± 11.04 and 36.21 ± 11.11, respectively). We found a significant difference between both sexes in the distal femoral and proximal tibial linear measurements where males had a significantly larger measurements in the anteroposterior [fAP: 60.97 ± 3.1 vs 54.78 ± 3.3 (*P* < 0.001), tAP: 46.89 ± 3.0 vs 41.35 ± 2.9 (*P* < 0.001)] and mediolateral [fML: 74.89 ± 3.2 vs 67.29 ± 3.7 (*P* < 0.001), tML: 76.01 ± 3.0 vs 67.26 ± 3.2 (*P* < 0.001)]. In contrast, the difference in the femoral and tibial aspect ratios was not significantly different, as shown in (Table 1).

**Table 1:** Gender difference in anthropometric knee measurements (in mm)

	Total (***n***=200)	Female (***n***=100)	Male (***n***=100)	***P*** value*
**Tibial mediolateral (tML)**				
**Mean** ± **SD** **(Range)**	71.62 ± 5.4 (61–83)	67.26 ± 3.2 (61–77)	76.01 ± 3.0 (68–83)	< 0.001
**Tibial anteroposterior (tAP)**				
**Mean** ± **SD** **(Range)**	44.14 ± 4.0 (36–54)	41.35 ± 2.9 (36–48)	46.89 ± 3.0 (41–54)	< 0.001
**Femoral ,mediolateral (fML)**				
**Mean ± SD** **(Range)**	71.09 ± 5.1 (59–85)	67.29 ± 3.7 (59–78)	74.89 ± 3.2 (66–85)	< 0.001
**Femoral anteroposterior (fAP)**				
**Mean** ± **SD** **(Range)**	57.88 ± 4.4 (42–68)	54.78 ± 3.3 (42–65)	60.97 ± 3.1 (53–68)	< 0.001
**Femoral aspect ratio (fAR)**				
**Mean** ± **SD** **(Range)**	1.23 ± 0.07 (1–1.5)	1.23 ± 0.08 (1–1.5)	1.23 ± 0.06 (1.1–1.5)	= 0.650*
**Tibial aspect ratio (tAR)**				
**Mean** ± **SD** **(Range)**	1.63 ± 0.09 (1.3–1.9)	1.63 ± 0.09 (1.3–1.9)	1.62 ± 0.09 (1.5–1.8)	= 0.932*

*Student’s *t* test was used to compare the means among groups

### Comparing the measurement of the study population with measurements of other ethnic groups (Table 2)

#### On the femoral side

The mean AP measurement in our population was smaller than the American and Chinese and larger than the Thai, Hispanic, and Indians. No difference was found compared to the Caucasians. The mean ML measurement in our population was smaller than the Korean population, larger than the Thai and Indian, and no difference was found compared to the American, Chinese, Caucasians, and Hispanic measurements. The AR was smaller than American, Thai, Hispanic, and Indians, larger than Chinese, and not different from the Caucasians.

**Table 2:** Summary of the morphometry of the proximal tibia and distal femur (in mm) in various studies

				(Morphometry of the proximal tibia)			(Morphometry of the distal femur)		
Authors	Population	Method	Study No.	t-ML	t-AP	t-AR	f-ML	f-AP	f-AR
Mensch et al. [12] I	American	Radiograph	30 cadavers 53 radiographic knees	80.3 ± 3.7 (M) 70.1 ± 2.8 (F) 74.9 ± 6.1 (C)	NR	NR	78.5 ± 7.1 (M) 70.5 ± 5.5 (F) 72.1 ± 6.6 (C)	78.5 ± 4.7 (M) 67.3 ± 4.9 (F) 68.4 ± 6.9 (C)	1.44 ± 0.1 (M) 1.43 ± 0.1 (F) 1.43 ± 0.1 (C)
Cheng et al. [19] II	Chinese	CT	172 non-arthritic	76.4 ± 2.8 (M) 68.8 ± 4.6 (F) 73.0 ± 4.6 (C)	51.3 ± 2.0 (M) 45.7 ± 1.9 (F) 48.8 ± 3.4 (C)	1.49 ± 0.05 (M) 1.51 ± 0.06 (F) 1.50 ± 0.05 (C)	74.4 ± 2.9 (M) 66.8 ± 3.1 (F) 71.0 ± 3.0 (C)	66.6 ± 2.4 (M) 61.0 ± 2.7 (F) 64.1 ± 2.7 (C)	1.12 ± 0.03 (M) 1.10 ± 0.02 (F) 1.11 ± 0.3 (C)
Chaichankul et al. [17] III	Thai	MRI	200 non-arthritic	74.4 ± 3.4 (M) 64.9 ± 3.5 (F) 68.8 ± 5.8 (C)	50.2 ± 3.1 (M) 43.2 ± 2.6 (F) 46.1 ± 4.4 (C)	NR	70.2 ± 3.9 (M) 59.9 ± 3.8 (F) 65.05 ± 3.85(C)	48.6 ± 3.7 (M) 43.3 ± 3.7 (F) 45.95 ± 3.7(C)	1.45 ± 0.1 (M) 1.39 ± 0.1 (F) 1.42 ± 0.1(C)
Li et al. [15] IV	Caucasian	CT	127 non-arthritic	79.4 ± 4.3 (M) 70.2 ± 2.7 (F) 74.8 3.5(C)	49.5 ± 2.9 (M) 45.2 ± 2.3 (F) 47.4 ± 2.6 (C)	1.61 ± 0.1 (M) 1.54 ± 0.1 (F) 1.6 ± 0.1(C)	74.6 ± 3.9 (M) 65.4 ± 1.4 (F) 70 ± 2.65(C)	59.6 ± 3.2 (M) 55.4 ± 2.8 (F) 57.5 ± 3(C)	1.25 ± 0.1 (M) 1.18 ± 0.1 (F) 1.22 ± 0.1(C)
Mcnamara et al. [20] V	Hispanic	MRI	500 non-arthritic	80.3 ± 4.0 (M) 69.8 ± 3.1 (F) 75.1 ± 3.6(C)	54.7 ± 3.3 (M) 47.1 ± 2.6 (F) 50.9 ± 3(C)	NR	77.2 ± 4.1 (M) 66.3 ± 3.0 (F) 71.8 ± 3.55(C)	49.9 ±3.8 (M) 45.6 ± 3.2 (F) 47.75 ± 3.5(C)	1.55 ± 0.1 (M) 1.46 ± 0.1 (F) 1.51 ± 0.1(C)
Mohan et al. [16] VI	Indian	MRI	100 non-arthritic	75.7 ± 4.3 (M) 65.5 ± 3.2 (F) 70.6 ± 3.8(C)	49.1 ± 3.9 (M) 43.3 ± 2.7 (F) 46.2 ± 3.3 (C)	1.55 ± 0.1 (M) 1.52 ± 0.07 (F) 1.54 ± 0.09(C)	73.7 ± 4.1 (M) 64.8 ± 3.4 (F) 69.3 ± 3.8(C)	57.5 ± 3.1 (M) 52.8 ± 3.1 (F) 55.2 ± 3.1(C)	1.28 ± 0.1 (M) 1.23 ± 0.1 (F) 1.26 ± 0.1(C)
Lim et al. [18] VII	Korean	MRI	115 non-arthritic	80.6 ± 6.3 (M) 70.0 ± 3.5 (F) 75.3 ± 4.9 (C)	NR	NR	81.5 ±5.7 (M) 76.7 ±3.7 (F) 79.1 ± 4.7(C)	NR	NR
Our Study VIII	Egyptian	MRI	200 non-arthritic	76.1 ± 3.0 (M) 67.3 ± 3.2 (F) 71.6 ± 5.4 (C)	46.9 ± 3.0 (M) 41.4 ± 2.9 (F) 44.2 ± 4.0 (C)	1.62 ± 0.1 (M) 1.63 ± 0.1 (F) 1.63 ± 0.1 (C)	74.9 ± 3.2 (M) 67.3 ± 3.7 (F) 71.1 ± 5.1 (C)	61.0 ± 3.1 (M) 54.8 ± 3.3 (F) 57.9 ± 4.4 (C)	1.23 ± 0.01 (M) 1.23 ± 0.02 (F) 1.23 ± 0.04 (C)
Comparing the results from previous studies with our results. *	VIII vs. I			< 0.001			= 0.013	< 0.001	< 0.001
	VIII vs. II			< 0.001	< 0.001	< 0.001	= 0.810	< 0.001	< 0.001
	VIII vs. III			< 0.001	< 0.001		< 0.001	< 0.001	< 0.001
	VIII vs. IV			< 0.001	< 0.001	< 0.001	= 0.003	= 0.228	= 0.002
	VIII vs. V			< 0.001	< 0.001		= 0.052	< 0.001	< 0.001
	VIII vs. VI			< 0.001	= 0.163	< 0.001	< 0.001	< 0.001	< 0.001
	VIII vs. VII			< 0.001			< 0.001		

*M* male, *F* female, *C* combined, *CT* computed tomography, *MRI* magnetic resonance imaging, *NR* not reported

*One sample *t* test was used

#### On the tibial side

The mean AP measurement was smaller than the Chinese, Thai, Caucasians, and Hispanic, with no difference from the Indians. The mean ML measurement was smaller than the Americans, Chinese, Caucasians, Hispanics, and Koreans but larger than the Thai and Indians. The AR was larger than the Chinese, Caucasians, and Indians.

### Comparing our results with Hafez et al. [23] study (Table 3) (where the population of the study came from the same geographic area as the population of the current study)

All the measurements in the current study were significantly smaller than what was reported in Hafez et al.’s study except for the tAR, which was greater in the current study.

**Table 3:** Comparison of the current study with Hafez et al.

				(Morphometry of the proximal tibia)			(Morphometry of the distal femur)		
Authors	Population	Method	Study knee No.	t-ML	t-AP	t-AR	f-ML	f-AP	f-AR
Hafez et al. [21]	Arabian	3D CT	124 (arthritic)	80.2 ± 4.6 (M) 72.9 ± 5.5 (F) 74.4 ± 6.0 (C)	52.5 ± 5.6 (M) 48.1 ± 3.9 (F) 48.9 ± 4.6 (C)	1.56 ± 0.1 (M) 1.52 ± 0.1 (F) 1.53 ± 0.1 (C)	78.5 ± 7.1 (M) 70.5 ± 5.5 (F) 72.1 ± 6.6 (C)	78.5 ± 4.7 (M) 67.3 ± 4.9 (F) 68.4 ± 6.9 (C)	1.43 ± 0.1 (M) 1.42 ± 0.1 (F) 1.43 ± 0.1 (C)
Our study	Egyptian	MRI	200 (non-arthritic)	76.1 ± 3.0 (M) 67.3 ± 3.2 (F) 71.6 ± 5.4 (C)	46.9 ± 3.0 (M) 41.4 ± 2.9 (F) 44.2 ± 4.0 (C)	1.62 ± 0.1 (M) 1.63 ± 0.1 (F) 1.63 ± 0.1 (C)	74.9 ± 3.2 (M) 67.3 ± 3.7 (F) 71.1 ± 5.1 (C)	61.0 ± 3.1 (M) 54.8 ± 3.3 (F) 57.9 ± 4.4 (C)	1.23 ± 0.01 (M) 1.23 ± 0.02 (F) 1.23 ± 0.04 (C)
*P* value				< 0.001*	< 0.001*	< 0.001*	< 0.001*	< 0.001*	< 0.001*

*M* male, *F* female, *C* combined

*One sample *t* test was used to compare the study mean vs the mean of other studies

### Comparing the measurement of the study population with geometric characteristics of seven TKA implants

The femoral and tibial AR of the current study population was higher than the femoral and tibial AR of all the implants used for comparison. This indicates a mismatch between the distal femoral and proximal tibial morphology and the sizes of the implants used in the comparison. For a given AP length, the implants’ ML dimension was smaller than the ML diameter of the knee, which may lead to under coverage.

#### Regarding femoral implants (Fig. 3)

With respect to the distal femoral implant dimensions, none of the seven TKA systems matched the distal femur's largest ML dimension in the current study population. However, all seven implants matched the smallest ML knee diameter. All the seven TKA systems matched the largest AP dimension of the distal femur in the Egyptian populations. None of the seven TKA systems matched the distal femur's smallest AP dimension (which was present in the female group).

**Fig. 3:**
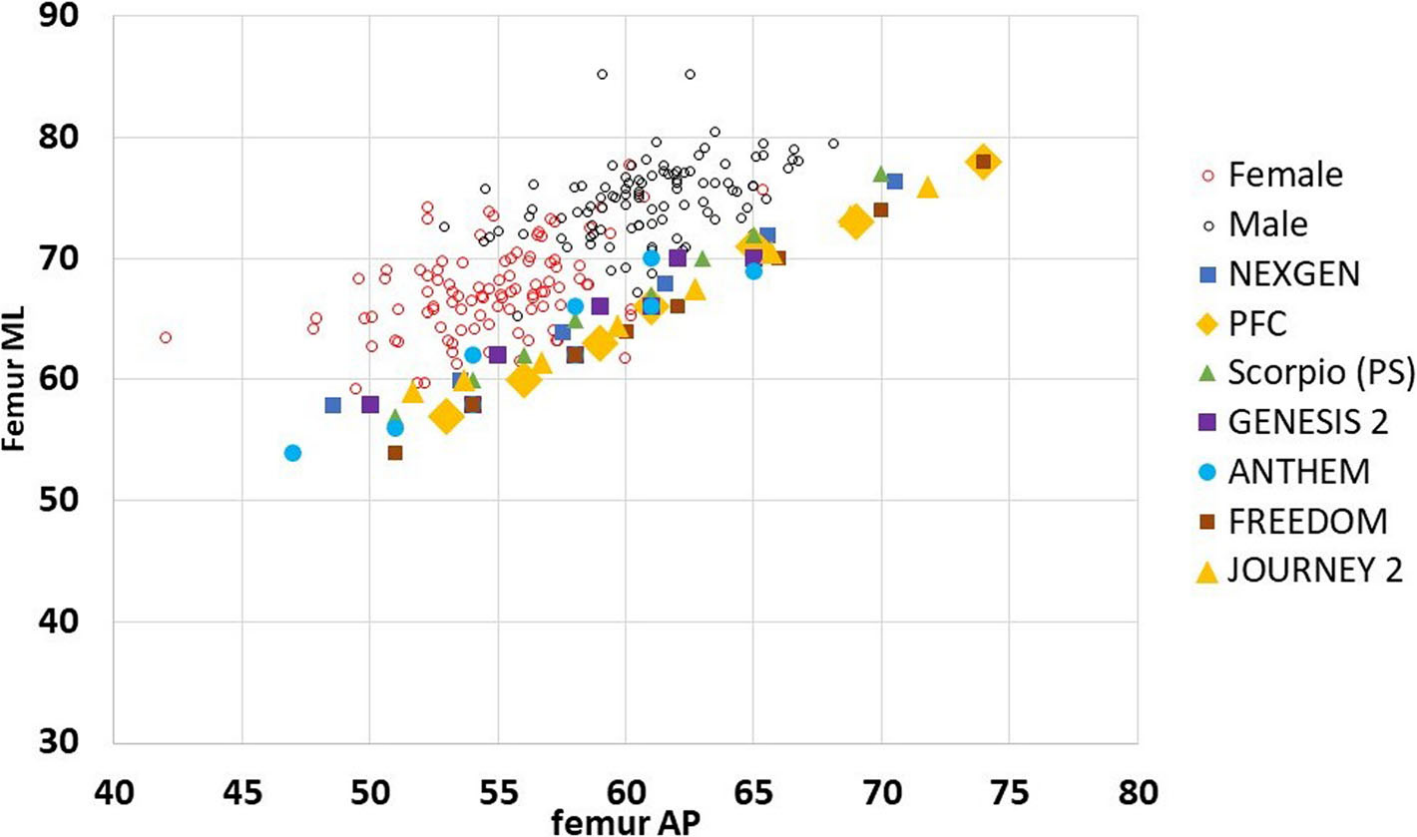
Graph showing correlations between the femoral anthropometric measurements and modern knee implant designs (fML, femoral mediolateral; fAP, femoral anteroposterior)

#### Regarding tibial implants (Fig. 4)

All the tibial implants could accommodate the ML dimensions of the proximal tibia in our population in the systems used for comparison; however, the smallest AP dimension (which was found in the female group) of the proximal tibia in the Egyptian population could not be matched with any of the seven TKA systems.

**Fig. 4:**
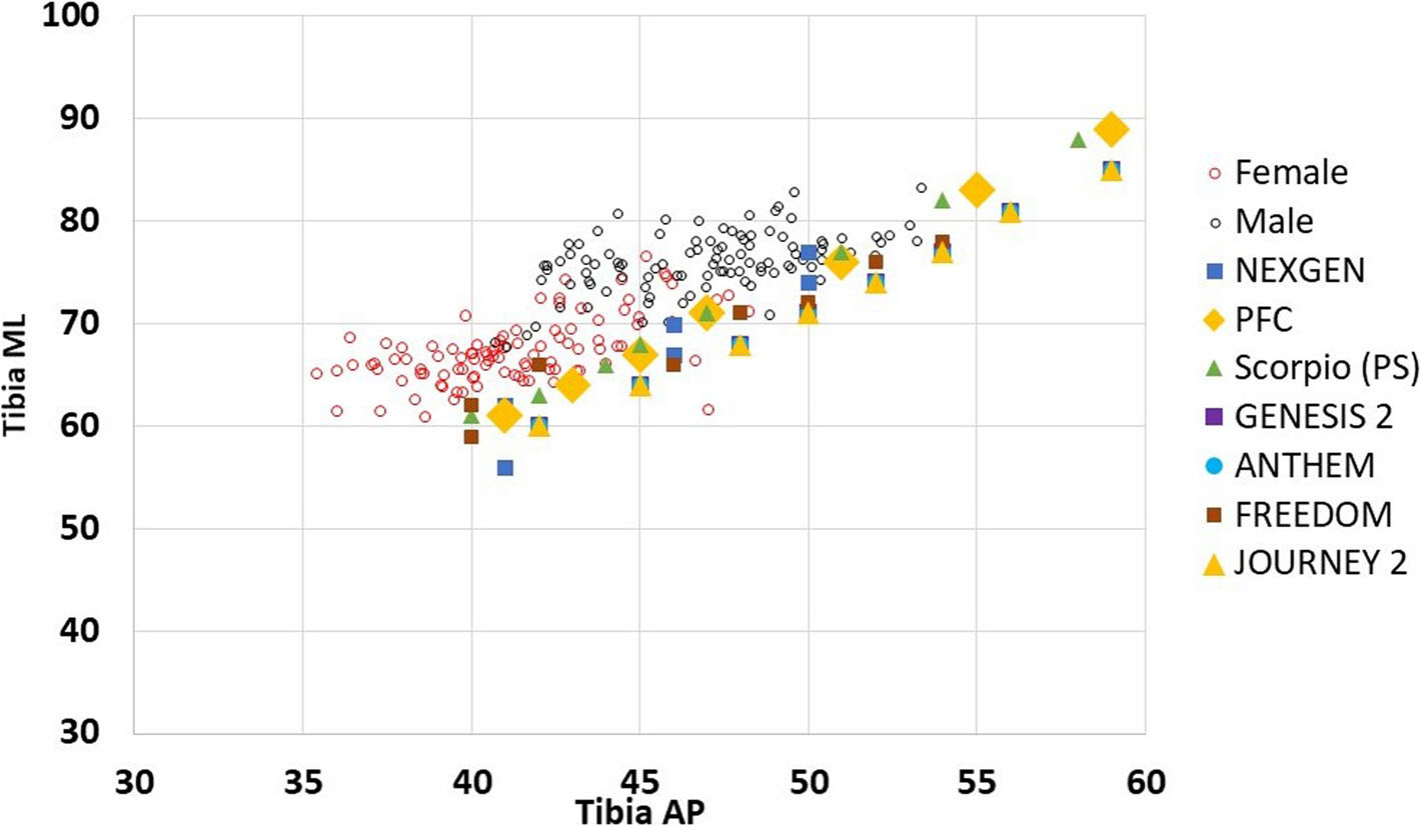
Graph showing correlations between the tibial anthropometric measurements and modern knee implant designs (tAP, tibial anteroposterior; tML, tibial mediolateral)

## Discussion

The present study's important findings were that in the Egyptian population, females had knees with smaller AP and ML dimensions in both the distal femur and proximal tibia compared to males, most of the current study linear measurements were different from the data reported on the anthropometric measurements of the various ethnic groups used for comparison (reported in detail in Table 2), and the Egyptian knees had a different shape compared to the commonly used TKA implant designs, where the smallest sizes in the Egyptian females could not match with any of the seven TKA designs used for comparison, including the three designs most commonly used in Egypt. To the best of our knowledge, this is the first study to report the anthropometric data of non-arthritic knees from Middle Eastern and North African populations.

TKA surgery aims to restore the function and biomechanics of the normal native knee. To get the perfect implant fit, the sizing is considered for the femoral and tibial components only after removing any osteophytes to reach the bony limits of the native knee [18]. To get accurate anthropometric data, the measurements should be performed on a normal knee model (either radiographic, CT, or MRI), as the arthritis process alters the anatomic dimensions [24]. In the current study, we used MRI scans to obtain the desired measurements; this technique was used by most previous studies reporting anthropometric knee evaluation [18–20, 22], as the values measured by MRI were reported to equal those measured intraoperatively by direct methods [27]. Moreover, MRI accounts for cartilage thickness, which corresponds to sizing techniques in most TKA systems [18].

The only study we could find reporting the measurement of an Arab population (which is similar to the current study population) was by Hafez et al. [23], where they reported the anthropometry of the arthritic Arabian knee by evaluating 126 CT scans. In concordance with our study, Hafez et al. found that ML and AP dimensions of the proximal tibia were significantly smaller in females than males. However, the measurements reported by Hafez et al.’s study were significantly larger than the current study except for the tAR. Although they subtracted the osteophytes from the measurements; still the presence of subchondral sclerosis and chondrocalcinosis alter the normal anatomy, thereby altering the normal knee anthropometry, which may affect the reliability of the measurements [24].

Some studies stressed the importance of matching TKA implant sizes and the native bony surfaces, both in the femur and the tibia, as adverse effects may result from unattended mismatch [28, 29]. Hitt et al. reported that lateral or medial overhang of the femoral or tibial implants could lead to soft tissue irritation, which may affect the soft tissue balance and be a source of chronic pain [30].

In the current study, we found that the AR (femoral and tibial) of both males and females are larger than all of the implants used for the comparison indicating that for a given AP dimension, the ML measurements in our population will be larger than that of the implant which will lead to the problem of under-coverage, to get a proper ML fit in our population the proper choice will be of an implant with a wider ML dimension, which if used from the current TKA implant designs will lead to increase in the AP dimension as well and subsequent overstuffing of the patellofemoral joint [31, 32].

Regarding the matching between the implants used for comparison and the anthropometric measurements of the current study, for the femoral component sizes, we identified subsets of Egyptians (especially females) with a small fAP diameter that the smallest TKA systems could not accommodate; however, in males, although the TKA designs covered all the AP diameters, males' femurs tended to be wider (larger ML diameter) than most components for a given AP size, which may lead to the problem of under coverage [33]. For the tibial component sizes, the tibias measured from Egyptian males matches well with the implants included in the comparison; however, in females, we found a subset with a small tAP diameter than all the tibial implants included in the comparison, which leads to the problem of tibial implant impingement on the posterior soft tissue structures [34].

The current study has some limitations; first, the study population came from a narrow geographical area, making the generalization of the results over other populations in our area difficult. Second, the measurements were taken on an ideal scenario tibial and femoral cuts; this may be untrue in real life in patients with severe deformities where the surgeon may have to take a bigger cut than the usually performed for straightforward cases; however, we believe that the measurements taken from normal knees should act as the foundation for designing newer implants for our population and the surgeon should take into his account the issue of extra bony cuts in cases with severe deformity during the pre-operative planning. Third, the study population's weight and height were not included in the analysis, which may correlate with the distal femur's dimensions and the proximal tibia. Fourth, measurements were driven from MRI images, which had been criticized for not showing a three-dimensional shape of knee joints, which may increase the incidence of AP and ML measurement errors; however, the method we used in the current study had been well documented and reported in previous studies handling the same issue. Last, comparing the results obtained from the current study with previous studies reported in the literature may be criticized for being unrealistic due to differences in measurement techniques, type of imaging modalities, and the state of the knee being arthritic or normal.

## Future perspectives

Further multicenter studies from different populations with larger numbers in our area are highly recommended; studies evaluating the possible effects of implants mismatch on the clinical outcomes are mandatory to back up the present finding aiming at providing special implants sizes according to various populations anthropometric measurements, and a discussion should be initiated with the manufacturers to take these studies and their findings in consideration when designing new implants.

## Conclusion

There is a significant difference in the knee anthropometric characteristics between males and females in our population, and some of the commonly used TKA implants in our area may not produce a perfect fit if used in our population. The current study results can provide insight for surgeons operating on knees from the same population of our study to select the most appropriate knee designs that will provide appropriate fit and coverage.

## Data Availability

All the data and materials were included within the manuscript.

## Abbreviations

AP: Anteroposterior
ML: Mediolateral
AR: Aspect ratio
TKA: total knee arthroplasty
MRI: Magnetic resonance imaging
PACS: Picture archiving and collecting system
f: Femoral
TEA: Trans-epicondylar axis
fML: Femoral mediolateral
fAP: Femoral anteroposterior
LDFC: Lateral distal femoral condyles
t: Tibial
tML: Tibial mediolateral
tAP: Tibial anteroposterior
ICC: Intraclass Correlation Coefficient
